# Circ-CCNB1 Modulates Trophoblast Proliferation and Invasion in Spontaneous Abortion by Regulating miR-223/SIAH1 axis

**DOI:** 10.1210/endocr/bqac093

**Published:** 2022-06-22

**Authors:** Meng-yu Jing, Lai-di Xie, Xi Chen, Ying Zhou, Meng-meng Jin, Wei-hua He, Di-min Wang, Ai-xia Liu

**Affiliations:** Department of Reproductive Endocrinology, Women’s Hospital, Zhejiang University School of Medicine, Hangzhou, 310006, PR China; Department of Reproductive Endocrinology, Women’s Hospital, Zhejiang University School of Medicine, Hangzhou, 310006, PR China; Department of Reproductive Endocrinology, Women’s Hospital, Zhejiang University School of Medicine, Hangzhou, 310006, PR China; Department of Reproductive Endocrinology, Women’s Hospital, Zhejiang University School of Medicine, Hangzhou, 310006, PR China; Department of Reproductive Endocrinology, Women’s Hospital, Zhejiang University School of Medicine, Hangzhou, 310006, PR China; Department of Obstetrics and Gynecology, First Affiliated Hospital, Zhejiang University College of Medicine, Hangzhou, 310003, PR China; Department of Reproductive Endocrinology, Women’s Hospital, Zhejiang University School of Medicine, Hangzhou, 310006, PR China; Department of Reproductive Endocrinology, Women’s Hospital, Zhejiang University School of Medicine, Hangzhou, 310006, PR China; Key Laboratory of Reproductive Genetics (Ministry of Education), Zhejiang University, Hangzhou, 310006, PR China

**Keywords:** spontaneous abortion, circ-CCNB1, trophoblast cells, miR-223, SIAH1

## Abstract

**Context:**

Spontaneous abortion (SA) is a common disorder in early pregnancy. Circular RNAs (circRNAs) have been reported to exert important regulatory effects on trophoblast function and embryo development.

**Objective:**

The aim of this study was to explore whether and how circRNAs regulate trophoblast function in SA during early pregnancy.

**Methods:**

Cell proliferation, 5-bromo-2-deoxyuridine (BrdU) staining, Transwell, immunofluorescence, Western blot, RNA pull-down, and dual luciferase reporter assays were performed to investigate the effect of circRNA cyclin B1 (circ-CCNB1) on trophoblast function in HTR-8/SVneo and JEG-3 cells.

**Results:**

An in vitro study demonstrated that upregulation of circ-CCNB1 significantly inhibited trophoblast proliferation and invasion compared with the controls using HTR-8/SVneo and JEG-3 cells, respectively. Moreover, miR-223 was downregulated in the villous tissues of patients with SA and was further predicted and shown to negatively interact with circ-CCNB1, which is involved in trophoblast proliferation and invasion. Using bioinformatics tools and subsequent RNA pull-down and dual luciferase assays, we found that miR-223 directly targets seven in absentia homolog-1 (SIAH1) and that upregulation of miR-223 decreased circ-CCNB1-induced SIAH1 expression levels in HTR-8/SVneo cells. Interestingly, upregulation of circ-CCNB1 suppressed trophoblast proliferation and invasion through inhibition of CCNB1 nuclear translocation induced by SIAH1. Downregulation of SIAH1 enhanced circ-CCNB1-suppressed CCNB1 nuclear protein expression in trophoblast cells.

**Conclusion:**

Circ-CCNB1 served as a modulator of trophoblast proliferation and invasion by sponging miR-223, thus forming a regulatory network of circ-CCNB1/miR-223/SIAH1 in modulating CCNB1 nuclear translocation, which enabled us to elucidate the molecular mechanisms involved in normal embryo implantation or in SA.

Spontaneous abortion (SA) is noninduced embryonic or fetal death before 20 weeks of gestation (1). The incidence of SA has been rising in recent years and accounts for nearly 10% to 15% of the total number of pregnancies worldwide (1, 2). A number of studies have shown that various factors induce SA, including fetal chromosomal abnormalities, advanced maternal age, previous early pregnancy loss, alcohol consumption, smoking, and cocaine use (3). Recently, several types of immune cells, such as macrophages, regulatory T cells (Tregs), and natural killer (NK) cells, have been found to participate in the pathology of SA (4, 5). Additionally, the interactions among immune cells, decidual stromal cells, and trophoblasts also act as an important regulatory network for various adverse pregnancy outcomes, including SA, preeclampsia, and intrauterine growth restriction (6). Despite the discovery of different mechanisms, the fundamental molecular mechanism of SA remains to be elucidated.

Previous studies have provided evidence of impaired invasion and proliferation of trophoblast cells in SA, suggesting that trophoblast cells play an important role in atypical embryo implantation during the pathophysiological development of SA (7-9). Evidence has demonstrated that the regulation of key biological processes does not depend only on classical transcriptional mechanisms but also includes other regulatory molecules, such as noncoding RNAs (10). MicroRNAs (miRNAs) are a class of ~22 nucleotide long noncoding RNAs that are widely involved in the posttranscriptional regulation of genes and a variety of regulatory pathways (11). Increasing evidence has shown that miR-223 is one of the key factors in the development and homeostasis of the immune system (12, 13) and may both inhibit or promote cell proliferation and invasion in various types of cells (14-16). Circular RNAs (circRNAs) are a new type of competitive endogenous noncoding RNA with regulatory effects, and they can act as competing endogenous RNAs (ceRNAs) and miRNA sponges, as well as competitors with RNA-binding proteins (RBPs), which regulate the transcription of circRNA-derived parental genes, encode proteins, and serve as molecular markers for diseases because of the strong stability and conserved expression of circRNA characteristics in the eukaryotic cytoplasm (17-19). Recently, circRNAs have been reported to regulate trophoblast function and embryo implantation (20, 21). A previous study showed that circRNA CCNB1 (circ-CCNB1) suppresses cell migration, invasion, and proliferation by inhibiting the formation of the CCNB1-Cdk1 complex in breast cancer cells (22). In tumor cells, the formation and activation of the CCNB1-Cdk1 complex initiates steps in mitosis, including condensation of chromosomes, assembly of spindle poles and breakdown of the nuclear envelope (23, 24). A recent study showed that circ-CCNB1 can bind to both CCNB1 and Cdk1 and prevent the interaction between CCNB1 and Cdk1 and the entry of these proteins into the nucleus (22).

Seven in absentia homolog-1 (SIAH1) is an E3 ubiquitin ligase that contains a RING finger structure that participates in ubiquitination and proteasome-mediated degradation of specific proteins (25, 26). SIAH1 is involved in various cell functions, including cell proliferation, invasion, and apoptosis, by mediating the ubiquitin–proteasome degradation process of target proteins (27, 28). In the present study, we found that circ-CCNB1 was significantly upregulated in villous tissues from patients with SA and might serve as a modulator of cell growth partly by sponging miR-223 and regulating downstream pathways in trophoblast cells. Hence, investigating the potential network of circ-CCNB1/miR-223 involved in trophoblast proliferation and invasion may help reveal the molecular pathways underlying the pathophysiological mechanisms of SA.

## Methods

### Clinical Specimens

Villous tissue samples from 16 patients with SA were collected from Women’s Hospital, School of Medicine, Zhejiang University, from 2018 to 2019. The diagnosis of SA was selected based on the patients’ medical history, including uterine malformations, high blood pressure, hyperthyroidism, and post test tube pregnancy results. Fourteen age-matched women with normal pregnancy at the same gestational age who underwent an elective abortion for nonmedical reasons were recruited as the control group. All participants had regular menstrual cycles, and their gestational age was estimated based on their last menstrual period. Villous tissue samples from SA patients and with normal pregnancy were taken through the cervix during dilatation and aspiration (29). After washing with 0.9% NaCl, samples were snap frozen in liquid nitrogen immediately and stored at −80 °C. The demographic characteristics of the studied women who experienced SA and normal pregnancy are shown in Table 1. Written informed consent was obtained from all participants prior to sample collection. This study was performed in accordance with the ethical standards in the Declaration of Helsinki, and the study protocol was approved by the Ethical Committee of the Women’s Hospital, School of Medicine, Zhejiang University, China.

**Table 1.** Characteristics of SA patients and control.

Variable	Control (n = 14)	SA (n = 16)	*P* value
Age (years)	28.36 ± 6.320	32.14 ± 4.312	0.0776
BMI (kg/m^2^)	21.3 ± 3.177	21.67 ± 2.653	0.6224
Gestational age (weeks)	7.34 ± 0.414	7.73 ± 0.628	0.0708
Progesterone (nmol/L)	44.96 ± 21.70	41.17 ± 18.70	0.8101
hCG (IU/L)	55186 ± 31020	20646 ± 15061	0.0185^**^
Alanine transaminase (U/L)	17.92 ± 21.14	13.06 ± 5.226	0.4321
Aspartate transaminase (U/L)	17.23 ± 10.94	15.24 ± 2.538	0.5314
Creatinine (μmol/L)	78.99 ± 7.882	69.01 ± 18.55	0.0584
Carbamide (mmol/L)	3.114 ± 1.057	3.506 ± 0.657	0.2555
Blastocyst (cm)	2.35 ± 0.812	2.593 ± 1.096	0.5311

Abbreviations: BMI, body mass index; hCG, human chorionic gonadotropin; SA, spontaneous abortion.

*P < 0.01.

### Cell Culture

The human extravillous trophoblast cell line HTR-8/SVneo and human choriocarcinoma cell line JEG-3 were acquired from American Type Culture Collection (ATCC, Manassas, VA, USA). Cells were cultured in high glucose Dulbecco’s modified Eagle’s medium (DMEM) supplemented with 10% fetal bovine serum (FBS, Gibco, NY, USA) at 37 °C in an atmosphere with 5% CO_2_.

### miRNA, circRNA, and Transfection

The miR-223 mimics, inhibitors, si-circ-CCNB1, si-SIAH1 and respective negative controls (NCs) were obtained from Sangon Biotech (Shanghai, China). The circ-CCNB1 overexpression vector (#GS0108) was obtained from Geneseed (Guangzhou, China). Cell transfection was performed using Lipofectamine 2000 reagent (Invitrogen, Carlsbad, CA, USA) according to the manufacturer’s instructions. In brief, 4 × 10^5^ HTR-8/SVneo cells or JEG-3 cells were seeded in 6-well plates for 16 hours. Then, 100 pmol miR-223 mimics, 100 pmol miR-223 inhibitors, 100 pmol si-circ-CCNB1, 100 pmol si-SIAH1, 100 pmol si-NC, or 8 μL Lipofectamine 2000 was diluted in 250 μL serum-free medium. After incubation for 5 minutes, the solutions were mixed gently and incubated for 20 minutes at room temperature. After 24 hours or 48 hours of transfection, the cells were harvested for further detection by green fluorescent protein (GFP) fluorescence.

### Subcellular Fractionation

HTR-8/SVneo cells were collected and used for nuclear and cytoplasmic protein extraction using an NE-PER Nuclear and Cytoplasmic Extraction Reagent kit (Life Technologies, Carlsbad, CA, USA). For nuclear and cytoplasmic RNA separation, the cells were collected and extracted using a PARIS kit (Life Technologies, Carlsbad, CA, USA).

### Fluorescence In Situ Hybridization

Fluorescence in situ hybridization (FISH) assays of HTR-8/SVneo cells were performed according to the manufacturer’s instructions. The Cy3-labeled circ-CCNB1 probes used in our study were designed and synthesized by GenePharma (Shanghai, China). The circ-CCNB1 probe was 5′TCAGCTCCATCTTCTGCATCCACATC3′. Briefly, the prepared cells were fixed with 4% paraformaldehyde containing 0.5% Triton X-100 for 20 minutes. The cells were incubated with the probes at 37 °C overnight, and then the cell nuclei were stained with DAPI (Beyotime). The staining results were observed in 4 fields using a fluorescence microscope (Leica, Wetzlar, Germany), and 3 replicates were performed.

### RNA Extraction and Quantitative Real-Time Reverse Transcription Polymerase Chain Reaction Analysis

Total RNA was isolated from fresh villous tissue and HTR-8/SVneo cells using the RNeasy Mini Kit (Qiagen, USA). RNA was quantified using spectrophotometric absorbance readings and treated with DNase I (Qiagen) to exclude DNA contamination. Then 1 µg of RNA was reverse transcribed with the Reverse Transcription Kit (Qiagen). An ABI PRISM 7500 real-time PCR system and miScript SYBR Green PCR Kit (Qiagen) were used to perform polymerase chain reactions. The amplification program consisted of 1 cycle of initial incubation at 50 °C for 2 minutes and 95 °C for 10 minutes, followed by 50 cycles of 95 °C for 15 seconds, 60 °C for 30 seconds, and 72 °C for 30 seconds, and a final extension at 72 °C for 5 minutes. The primers used in the study are listed in Table S1. GAPDH was used as an internal control for mRNA. U6 was used as an internal control for miRNA. Relative RNA expression was calculated by the standard 2^-ΔΔCt^method.

### Western Blot

After transfection, HTR-8/SVneo cells or JEG-3 cells were treated in lysis buffer. The protein concentration was quantified by a BCA Protein Assay Kit (Beyotime, China). Subsequently, the proteins were transferred to nitrocellulose membranes, blocked with 2% bovine serum albumin (BSA), and incubated with a specific primary antibody at 4 °C overnight. The membranes were washed and incubated with secondary antibody for 2 hours at room temperature. Finally, the protein bands were visualized using a chemiluminescence detection system. The antibodies against SIAH1 (#ab2237, Abcam, Cambridge, UK, RRID: AB_2270373), CCNB1 (ab32053, Abcam, Cambridge, UK, RRID: AB_731779), GAPDH (#3683, Cell Signaling Technology, Danvers, MA, USA, RRID:AB_1642205), tubulin (#56739, Cell Signaling Technology, Danvers, MA, USA, RRID: AB_2799519), proliferating cell nuclear antigen (PCNA) (ab15497, Abcam, Cambridge, UK, RRID: AB_2160360), and secondary anti-IgG (ab205718, Abcam, Cambridge, UK, RRID:AB_2819160) antibodies were used to determine the protein expression levels. ImageJ (NIH) was used to quantify the proteins.

### Northern Blot

Northern blot was performed as previously described (30). Briefly, RNA prepared from HTR-8/SVneo cells was electrophoresed and transferred onto a positively charged nylon membrane (GE Healthcare, Shanghai, China). The membrane was crosslinked and hybridized with 3′-digoxigenin (DIG) labeled probes overnight (miR-223 probe: ACGGAGCGTAACACTGCAGAAG). Then, the blots were detected using the chemiluminescent detection system.

### Cell Proliferation Assay

Cell proliferation was examined using a Cell Counting Kit-8 (CCK-8) assay kit (Dojindo, Japan). Briefly, HTR-8/SVneo cells or JEG-3 cells were transfected with circ-CCNB1 vector or control vector using Lipofectamine 2000 reagent. After 24 hours or 48 hours of transfection, 10 μL CCK-8 solution was added to the cells, which were then incubated for 2 hours. The absorbance at 450 nm was measured with a microplate reader (ELX800, BioTeK, USA).

### 5-Bromo-2-Deoxyuridine Staining Assay

HTR-8/SVneo cells (4 × 10^5^) were treated with circ-CCNB1 vector or control vector for 48 hours, incubated with 10 µM 5-bromo-2-deoxyuridine (BrdU) (Beyotime, Shanghai, China) for 30 minutes at 37 ºC in the dark, and then washed with phosphate-buffered saline (PBS) and fixed in cold 70% ethanol for 30 minutes. The fixed cells were treated with 1 M HCl and blocked with 1% BSA (Beyotime) at room temperature for 1 hour, followed by a monoclonal antibody against BrdU (1:200; #ab152095, Abcam, RRID: AB_2813902) for 1 hours and Alexa Fluor 594 secondary antibody (1:1000; #ab150084 Abcam, RRID: AB_2734147) for 30 minutes at room temperature. DAPI (Beyotime) was used to stain the nuclei. Images were visualized in 3 randomly selected areas with 3 experimental replicates using an inverted fluorescence microscope (Leica, Wetzlar, Germany).

### Cell Invasion Assays

The cell invasion ability was analyzed using Matrigel-coated Transwell cell culture chambers (BD Matrigel Invasion Chamber, BD Biosciences, USA) with an 8-μm pore size. Briefly, 1 × 10^4^ HTR-8/SVneo cells or JEG-3 cells were seeded with Matrigel in the upper chambers. The lower chambers contained DMEM with 10% fetal bovine serum. After culturing at 37 °C and 5% CO_2_ for 48 hours, cells on the upper side of the chamber invaded the lower chamber. The invaded cells were stained using 0.1% crystal violet. The invaded cell number was counted in 3 randomly selected areas under an inverted light microscope (Olympus, Tokyo, Japan), and 3 experimental replicates were performed.

### Cell Cycle Analysis

HTR-8/SVneo cells were seeded and transfected with circ-CCNB1 vector or vector control for 48 hours at 37 °C. The treated cells were washed with PBS and fixed in cold 70% ethanol for 30 minutes. After washing with PBS, the cells were incubated with 400 µL propidium (PI) for 30 minutes in the dark at room temperature. The cellular DNA content was measured by flow cytometry with the aim of revealing the cell distribution within the major phases of the cell cycle. The DNA content was detected using a flow cytometer (BD Biosciences), and the cell cycle was analyzed using FlowJo 7.6.1 software (FlowJo LLC).

### RNA Pull-Down Analysis

The biotin (bio)-labeled circ-CCNB1 and control probe, bio-labeled miR-223 and bio-labeled negative control (NC) (GenePharma, Shanghai) were incubated with magnetic beads (Life Technologies, Carlsbad, CA, USA) at 37 °C for 4 hours. For the circ-CCNB1/miR-223 pull-down analysis, circ-CCNB1-overexpressing and control HTR-8/SVneo cells were lysed and incubated with the beads at 4 °C overnight. For the miR-223/SIAH1 pull-down analysis, 4 × 10^5^ cells were transfected with bio-miR-223 or bio-NC using Lipofectamine 2000 reagent. The cells were lysed and incubated with magnetic beads (Life Technologies, Carlsbad, CA, USA) at 4 °C overnight. After washing with wash buffer, the SIAH1 in the pull-down was determined using Western blot and quantitative reverse-transcriptase polymerase chain reaction (qRT-PCR) assays.

### Dual Luciferase Assays

The fragments containing the wild-type (WT) and mutant (Mut) circ-CCNB1 and SIAH1 genes were cloned into the psiCHECK-2 vector (Promega, Madison, WI). For the circ-CCNB1-miR-223 luciferase assays, 4 × 10^5^ HTR-8/SVneo cells were cotransfected with miR-223 mimics and the circ-CCNB1 luciferase reporter. For the miR-223-SIAH1 luciferase assay, 4 × 10^5^ HTR-8/SVneo cells were transfected with miR-223 mimics combined with the (WT and Mut) SIAH1-3′UTR-luciferase reporter. The luciferase activity of the cells was detected using a Dual Luciferase Reporter Assay System (Promega).

### Histological Analysis

Paraffin-embedded slides of villous tissue were dewaxed in xylol for 20 minutes and rinsed with 100% ethanol for the staining process. Each slide was incubated with Ki-67 antibody (1:100, #2642-1, Abcam, RRID: AB_1580751) for 24 hours at 4 °C. Immunostaining was visualized with the DAB staining system (Beyotime). HTR-8/SVneo cells were fixed and permeabilized with 4% paraformaldehyde containing 0.5% Triton X-100 for 20 minutes. Fixed cells were washed and blocked in 1% BSA for 1 hour and incubated with anti-SIAH1 (1:200, #ab2237, Abcam, RRID: AB_2270373) and anti-CCNB1 (1:200, #ab32053, Abcam, RRID: AB_731779) antibodies at 4 °C overnight. The cells were washed with PBS and incubated with Alexa Fluor 488 antibody (1:1000; #ab150077, Abcam, RRID: AB_2630356). The nuclei were stained with DAPI (Beyotime). Images were visualized using an inverted fluorescence microscope (Leica, Wetzlar, Germany).

### Statistical Analysis

The data were analyzed using SPSS 17.0 software (SPSS Inc., Chicago, IL, USA). The data are presented as the mean ± SD of at least 3 independent experiments. Significant differences were calculated using Student’s *t* test or 1-way ANOVA with Tukey’s test. Statistical significance was defined as *P *< 0.05.

## Results

### Upregulation of circ-CCNB1 in Patients With SA

**Figure 1.**
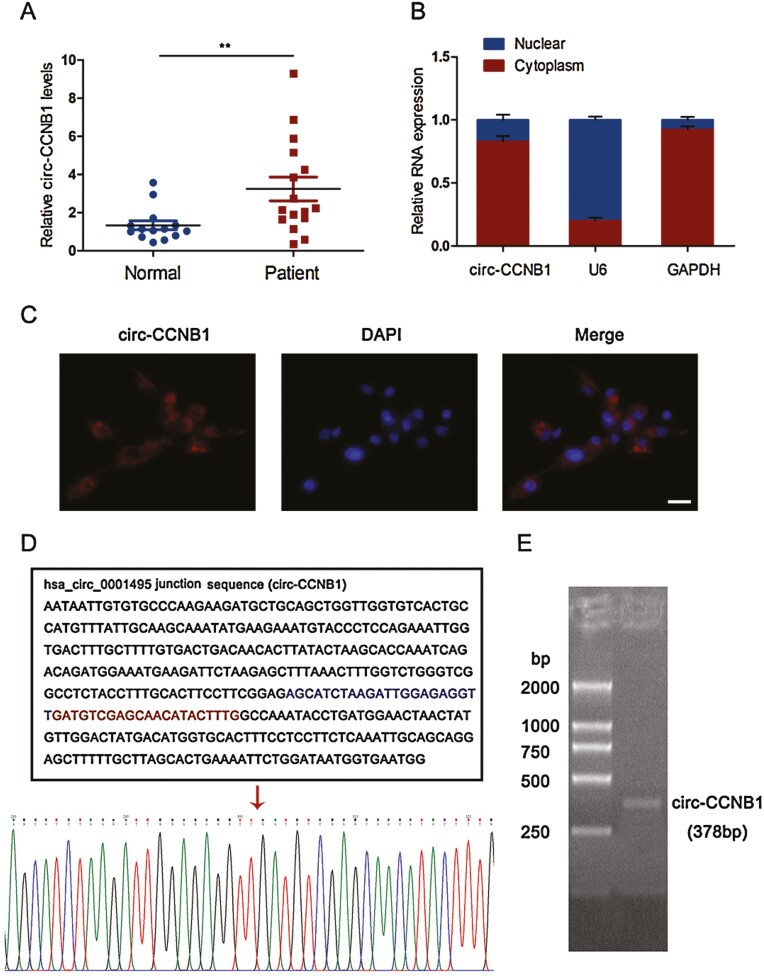
Identification and characterization of circ-CCNB1 in SA patients. (A) The expression levels of circ-CCNB1 were determined in villous tissues of SA (n = 16) and normal control (n = 14), **P < 0.01, Student’s *t* test. (B) The cellular distribution of circ-CCNB1 was analyzed by cellular RNA fractionation assays. GAPDH and U6 were used as cytoplasmic and nuclear positive controls, respectively. The data were presented for 3 independent experiments. (C) The cellular distribution of circ-CCNB1 was analyzed by FISH in HTR-8/SVneo cells. Red indicated circ-CCNB1. Nuclei were stained with DAPI, Scale bar, 20 μm. The spliced sequences of circ-CCNB1 were validated by Sanger sequencing in HTR-8/SVneo cells (D) and agarose gel electrophoresis (E). The spliced length of circ-CCNB1 is 378 bp. The red arrow indicated the back splice site.

Circ-CCNB1 is a regulatory circRNA involved in cell proliferation and invasion (31), and it might be associated with the aberrant function of trophoblast cells during embryo implantation. As shown in Fig. 1A, the expression levels of circ-CCNB1 in patients with SA were significantly increased compared with those in the normal controls. To investigate the effect of circ-CCNB1 on trophoblast function, the cellular RNA fractionation and distribution of circ-CCNB1 were explored in HTR-8/SVneo cells. We found that circ-CCNB1 was predominantly distributed in the cytoplasm of trophoblast cells based on the cellular RNA fractionation (Fig. 1B) and FISH assay results (Fig. 1C). The Sanger sequencing results confirmed the spliced sequence in the qRT-PCR product of circ-CCNB1 in HTR-8/SVneo cells (Fig. 1D). Moreover, the sequences of circ-CCNB1 were further validated by agarose gel electrophoresis. The spliced mature sequence length of circ-CCNB1 was 378 base pairs (bp) (Fig. 1E).

### Circ-CCNB1 Suppressed Trophoblast Cell Proliferation In Vitro

**Figure 2.**
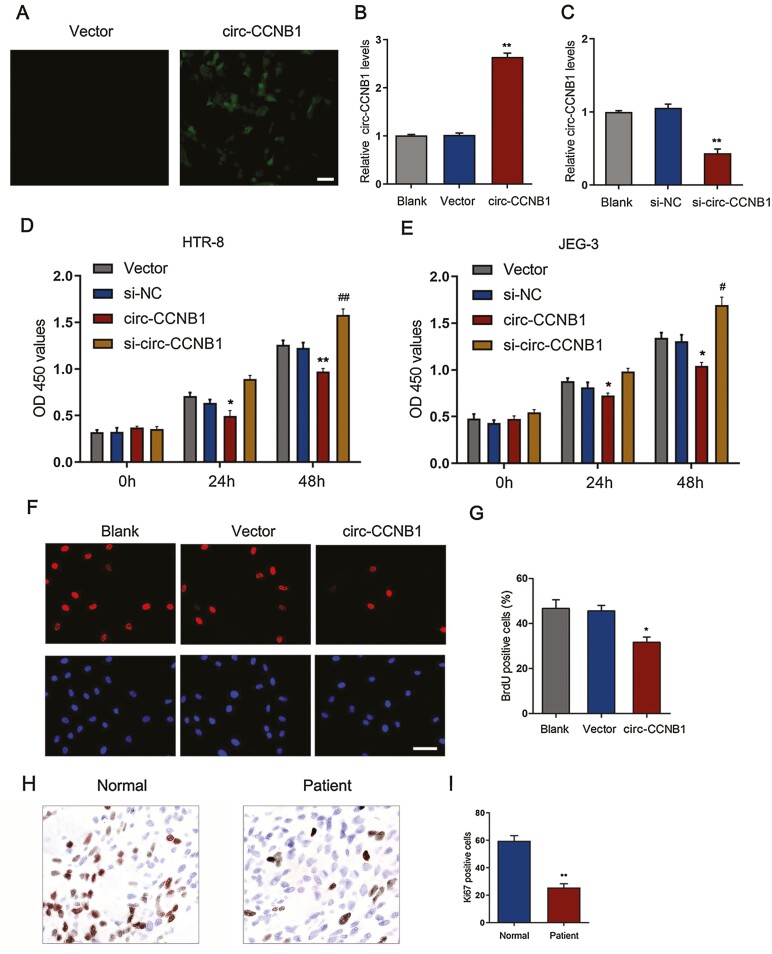
Circ-CCNB1 suppressed trophoblast proliferation in vitro. Overexpression of circ-CCNB1 by transfection of circ-CCNB1 vectors in HTR-8/SVneo cells. Overexpression of circ-CCNB1 was determined by the GFP positive cells (A) or circ-CCNB1 expression levels (B), scale bar: 50 μm, **P < 0.01 VS. Vector group. (C) The expression levels of circ-CCNB1 were determined following treatment with si-circ-CCNB1, **P < 0.01 VS. si-NC group. The cell proliferation was estimated by overexpression of circ-CCNB1 or by downregulation of circ-CCNB1 for 0, 24 and 48 hours in HTR-8/SVneo (D) or JEG-3 cells (E), *P < 0.05 vs Vector group, **P < 0.01 vs Vector group. (F) Immunofluorescence analysis of BrdU incorporation in HTR-8/SVneo cells transfected with circ-CCNB1 vector or vector control for 48h. BrdU was stained with Alexa Fluor 594 conjugated antibodies (red). Cell nuclei were visualized by DAPI staining (blue), scale bar: 50 μm. (G) Quantification of BrdU staining cells was determined (n = 3), *P < 0.05 vs Vector group. (H) Expression of Ki67 was determined in villous tissue of SA patients or normal pregnancy (400 × magnification). (I) Quantification of Ki67 positive cells was determined in villous tissue of SA patients or normal pregnancy (n = 3). **P < 0.01. The data were presented for 3 independent experiments. The statistical differences were calculated by 1-way ANOVA analysis of variance with Tukey’s test.

To investigate the effect of circ-CCNB1 on the regulation of trophoblast function, circ-CCNB1 was upregulated by transfection of the circ-CCNB1 overexpression vector in HTR-8/SVneo cells. The transfection efficiency was evaluated by GFP fluorescence (Fig. 2A). Moreover, the circ-CCNB1 overexpression levels were significantly enhanced by treatment with the circ-CCNB1 overexpression vector (Fig. 2B) but decreased by transfection with si-circ-CCNB1 in HTR-8/SVneo cells (Fig. 2C). To determine the influence of circ-CCNB1 on the function of trophoblast cells, cell proliferation was tested in HTR-8/SVneo and JEG-3 cells 24 hours and 48 hours after transfection with the circ-CCNB1 vector or si-circ-CCNB1. The results showed that overexpression of circ-CCNB1 led to a significant decrease in cell proliferation at 24 hours and 48 hours compared with vector group in HTR-8/SVneo (Fig. 2D) and JEG-3 cells (Fig. 2E) while transfection with si-circ-CCNB1 significantly increased cell proliferation at 48 hours in HTR-8/SVneo cells (Fig. 2D) and JEG-3 cells (Fig. 2E). Moreover, immunofluorescent staining of BrdU confirmed that overexpression of circ-CCNB1 markedly inhibited cell proliferation in HTR-8/SVneo cells (Fig. 2F and 2G). The number of Ki67-positive cells in the villous tissues of SA patients was significantly decreased compared with that in the normal controls (Fig. 2H and 2I), suggesting that trophoblast cell proliferation was inhibited in SA patients.

### Circ-CCNB1 Inhibited Trophoblast Cell Invasion and Induced Cell Cycle Arrest in HTR-8/SVneo Cells

**Figure 3.**
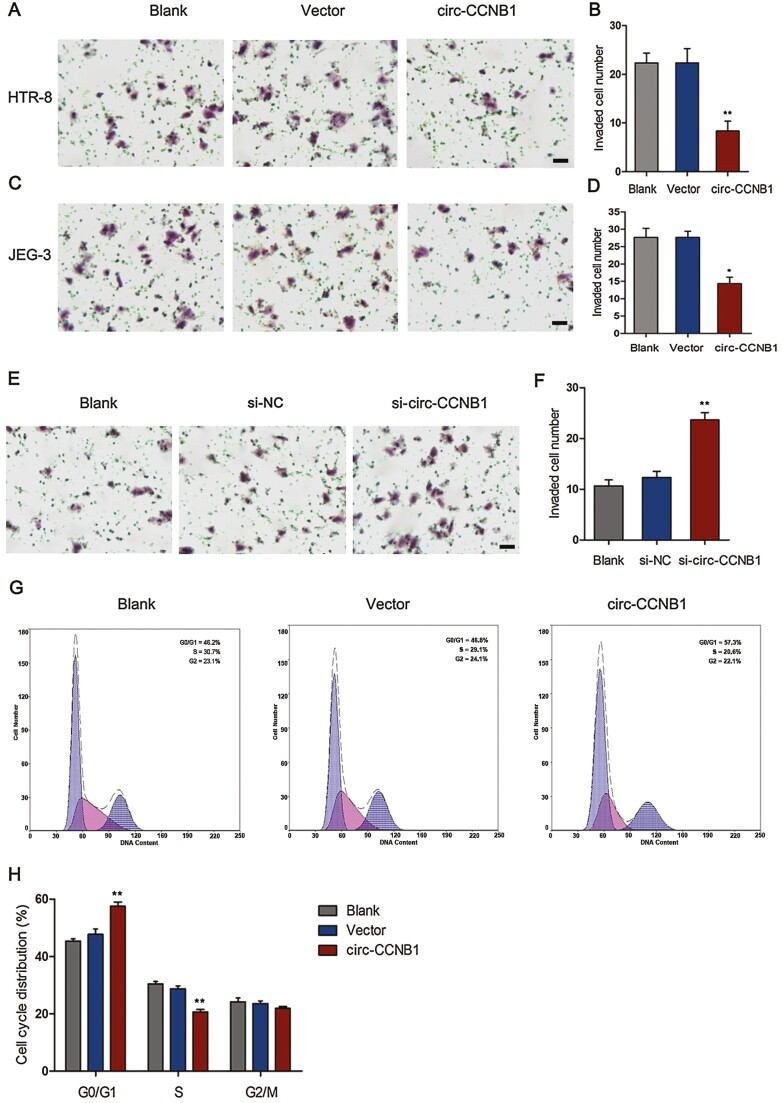
Circ-CCNB1 inhibited trophoblast cell invasion and arrested cell cycle at G0/G1 in HTR-8/SVneo cells. The cell invasion was determined by overexpression of circ-CCNB1 for 48 hours in HTR-8/SVneo (A) or JEG-3 cells (C). Scale bar, 50 μm. Quantification of the cell invasion in HTR-8/SVneo (B) or JEG-3 cells (D), *P < 0.05 vs Vector group, **P < 0.01 vs Vector group. (E, F) The cell invasion and quantification were evaluated by downregulation of circ-CCNB1 for 48 hours in HTR-8/SVneo cells, **P < 0.01 VS. Vector group, scale bar: 50 μm. (G, H) Distribution of cell cycle for HTR-8/SVneo cells was detected after treated with circ-CCNB1 vector or vector control for 24 hours, **P < 0.01 vs Vector group. The data were presented for 3 independent experiments. The statistical differences were calculated by 1-way ANOVA analysis of variance with Tukey’s test.

To further explore the effect of circ-CCNB1 on the regulation of trophoblast function, cell invasion was determined in HTR-8/SVneo and JEG-3 cells after transfection with the circ-CCNB1 vector at the indicated time points. The invaded cells were evaluated by 0.1% crystal violet staining. The results showed that circ-CCNB1 overexpression led to a significant decrease in the invaded cell number in the HTR-8/SVneo (Fig. 3A and 3B) and JEG-3 cells (Fig. 3C and 3D) compared with those in the control group, whereas circ-CCNB1 downregulation by the si-circ-CCNB1 treatment led to a significant increase in the invaded cell number in the HTR-8/SVneo cells compared with those in the control group (Fig. 3E and 3F). To explore whether circ-CCNB1-suppressed trophoblast function was associated with cell cycle arrest, the cell cycle distribution of HTR-8/SVneo cells was determined based on a flow cytometry assay. The cellular DNA content was detected by flow cytometry to reveal the phases of the cell cycle. As shown in Fig. 3G and 3H, overexpression of circ-CCNB1 led to a significant increase in the percentage of HTR-8/SVneo cells in the G0/G1 phase and a significant decrease in the percentage of HTR-8/SVneo cells in the S phase compared with the control group. These results suggested that circ-CCNB1 arrested the cell cycle at G0/G1 in HTR-8/SVneo cells.

### Circ-CCNB1 Regulated Trophoblast Cell Proliferation and Invasion by Sponging miR-223

**Figure 4.**
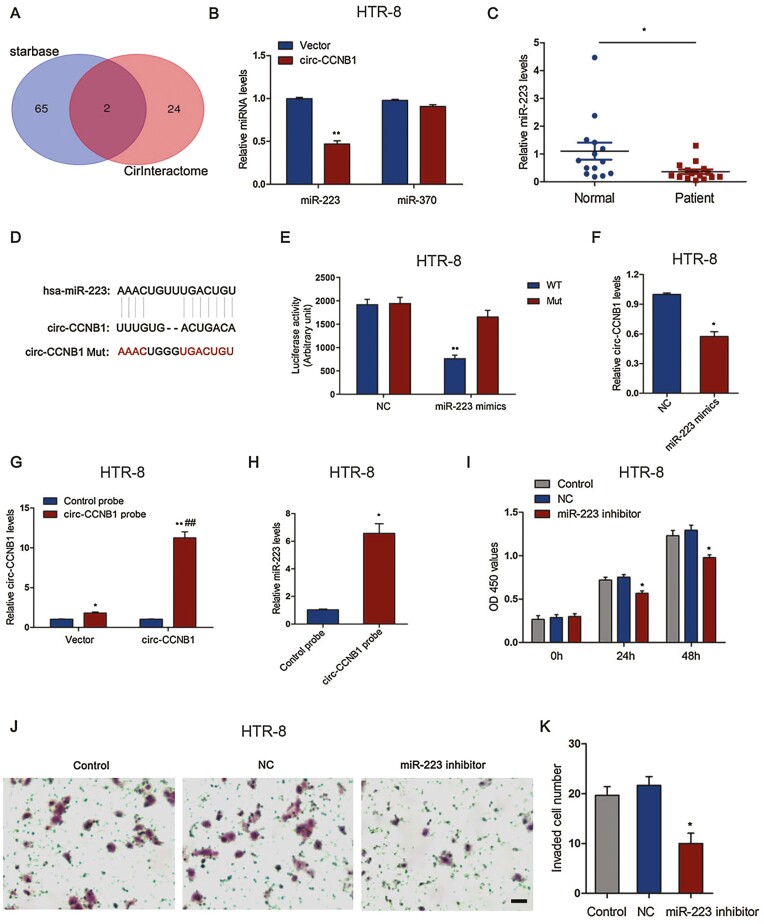
Circ-CCNB1 regulated trophoblast cell proliferation and invasion through sponging miR-223. (A) Venn diagram showing overlapped miRNAs that were predicted interaction with circ-CCNB1 by the starBase and CircInteractome analysis, respectively. (B) The relative miR-370 and miR-223 expression levels were determined following treatment with circ-CCNB1 in HTR-8/SVneo cells. (C) The expression levels of miR-223 were determined in villous tissues of SA (n = 16) and normal control (n = 14), *P < 0.05. (D) The targeted sequence of miR-223 from the target prediction program. (E) HTR-8/SVneo cells were co-transfected with miR-223 mimics or mimics control (NC) and/or WT/Mut of circ-CCNB1 luciferase reporter. Luciferase reporter assays were used to detect luciferase activity, **P < 0.01 vs NC group. (F) The relative expression levels of circ-CCNB1 were determined following treatment with miR-223 mimics or NC in HTR-8/SVneo cells, *P < 0.05. (G) The pull-down efficiency of circ-CCNB1 or control probe was evaluated following transfection with circ-CCNB1 or empty vectors in HTR-8/SVneo cells, *P < 0.05, **P < 0.01 vs Control probe, ^##^*P* < 0.01 VS. Vector group. (H) The pull-down assay was used to determine the interaction of circ-CCNB1 and miR-223 in HTR-8/SVneo cells, *P < 0.05. (I) The cell proliferation was estimated by treatment with miR-223 inhibitor or inhibitor control (NC) for 0, 24, and 48 hours in HTR-8/SVneo cells, *P < 0.05 vs NC group. (J, K) The cell invasion and quantification were evaluated by treatment with miR-223 inhibitor or NC for 48 hours in HTR-8/SVneo cells, *P < 0.01 VS. NC group, scale bar: 50 μm. The data were presented for 3 independent experiments. The statistical differences were calculated by Student’s *t* test or 1-way ANOVA analysis of variance with Tukey’s test.

To explore the detailed mechanism by which circ-CCNB1 regulates trophoblast proliferation and invasion and the cell cycle, tentative miRNAs were predicted by the target prediction programs (CircInteractome and starBase). The prediction programs showed 2 overlapping miRNAs were targeted by circ-CCNB1 (Fig. 4A). The relative expression levels of miR-370 and miR-223 were evaluated by the circ-CCNB1 treatment, and the results showed that miR-223 was significantly suppressed after overexpression of circ-CCNB1 in HTR-8/SVneo cells (Fig. 4B). Consistently, the levels of miR-223 in patients with SA were significantly lower than those in normal controls (Fig. 4C), which was negatively associated with the circ-CCNB1 levels in patients with SA. The negative coefficient between miR-223 and circ-CCNB1 is shown in Supplementary Fig. 1C (32). A previous study showed that the miR-223 expression levels were dramatically decreased in patients with recurrent pregnancy loss (33), which indicated that miR-223 might be involved in the pathophysiological mechanisms of SA. To further verify the relationship between circ-CCNB1 and miR-223, a luciferase reporter assay was performed in HTR-8/SVneo cells following treatment with miR-223 mimics and/or WT/Mut circ-CCNB1 luciferase reporters. The sequence of miR-223 was predicted to interact with circ-CCNB1 from the target analysis results (Fig. 4D). Moreover, the luciferase activity of circ-CCNB1 WT reporters was significantly reduced in the presence of miR-223 mimics compared with the control, whereas the luciferase activity was barely changed in the circ-CCNB1 Mut reporters (Fig. 4E). The expression levels of circ-CCNB1 significantly decreased following treatment with miR-223 mimics (Fig. 4F). To further determine the interaction between circ-CCNB1 and miR-223, a biotin-labeled circ-CCNB1 probe pull-down assay was performed in the HTR-8/SVneo cells. The results showed that the pull-down efficiency of circ-CCNB1 was significantly enhanced in the circ-CCNB1-overexpressing cells compared with the vector control and probe control groups (Fig. 4G). The expression levels of miR-223 were significantly enriched in the circ-CCNB1 probe-treated precipitates compared with the control (Fig. 4H). To explore the effect of miR-223 on trophoblast cell proliferation and invasion, HTR-8/SVneo cells were treated with a miR-223 inhibitor at the indicated time points. The results showed that the miR-223 inhibitor treatment resulted in a significant decrease in cell proliferation at 24 hours and 48 hours in the HTR-8/SVneo cells (Fig. 4I). In addition, the miR-223 inhibitor treatment markedly decreased the invaded cell numbers when compared with the control (Fig. 4J and 4K).

### MiR-223 Targeted SIAH1 and Reversed circ-CCNB1-Enhanced SIAH1 Expression in HTR-8/SVneo Cells

**Figure 5.**
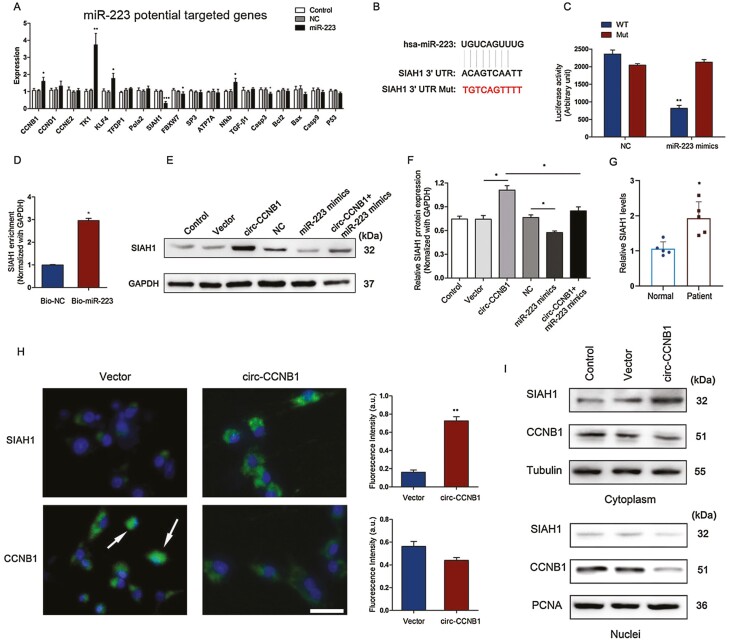
Circ-CCNB1 inhibited CCNB1 nuclear translocation in trophoblast cells HTR-8/SVneo cells. (A) The expression levels of miR-223 potential targeted cyclin-related genes, ubiquitin ligases, and apoptotic factors were determined following treatment with miR-223 mimics or NC in HTR-8/SVneo cells. (B) Prediction of miR-223 binding site in SIAH1 3′-UTR. (C) Luciferase reporter assays were performed to detect the luciferase activity in HTR-8/SVneo cells after co-transfection with the miR-223 mimics or NC and/or SIAH1 WT/Mut of 3′-UTR luciferase reporters. **P < 0.01 vs NC group. (D) Enrichment of SIAH1 transcripts in Bio-miR-223 pull-down mRNA in HTR-8/SVneo cells, *P < 0.05. (E, F) The protein expression levels and quantification of SIAH1 were determined following treatment with circ-CCNB1 vector and/or miR-223 mimics in HTR-8/SVneo cells, *P < 0.05. (G) The expression levels of SIAH1 were determined in SA patients or normal pregnancy (n = 5), *P < 0.05. (H) The immunofluorescence of SIAH1 was performed in HTR-8/SVneo cells. The circ-CCNB1-transfected HTR-8/SVneo cells were subjected to immunofluorescent staining. Circ-CCNB1 decreased nuclear localization of CCNB1, scale bar: 25 μm. The white row represented nuclear localization of CCNB1 in HTR-8/SVneo cells. Intensify quantification of SIAH1 and CCNB1 florescence in trophoblast cells at 48 hours after circ-CCNB1 transfection, n = 3 per group, **P < 0.01. (I) The cytoplasmic and nuclear SIAH1 and CCNB1 protein expression levels were determined following treatment with circ-CCNB1. The data were presented for 3 independent experiments. The statistical differences were calculated by Student’s t test or one-way ANOVA analysis of variance with Tukey’s test.

To investigate the downstream signaling pathway of miR-223 involved in trophoblast function, the expression levels of potential miR-223-targeted cyclin-related genes, ubiquitin ligases, transcription factors, and apoptotic factors were determined following treatment with miR-223 mimics in HTR-8/SVneo cells. The results showed that the miR-223 mimic treatment dramatically increased the expression levels of the cyclin-related genes CCNB1, TK1, and KLF4 and the transcription factor NF-κB while significantly decreasing the expression levels of the E3 ubiquitin ligases SIAH1 and FBXW7 as well as the apoptotic factor caspase-3 (Fig. 5A). Moreover, the binding site of the SIAH1 3′-UTR was shown to be targeted by miR-223 (Fig. 5B). The luciferase activity of SIAH1 WT 3′-UTR reporters was significantly decreased in the presence of miR-223 mimics compared with the mimic control, while it was not significantly changed in the SIAH1 Mut 3′-UTR reporters (Fig. 5C). To further determine the interaction between miR-223 and SIAH1, a biotin-labeled miR-223 pull-down assay was performed in the HTR-8/SVneo cells. The precipitated products were also used to test the mRNA expression levels of SIAH1. We found that biotin-labeled miR-223 precipitated significantly higher levels of SIAH1 mRNA compared with the control (Fig. 5D). In addition, we also found that the protein expression levels of SIAH1 were significantly enhanced by overexpression of circ-CCNB1 compared with the control but were decreased in the combined treatment of miR-223 mimics and circ-CCNB1 compared with the circ-CCNB1 overexpression group (Fig. 5E and 5F). Consistently, the SIAH1 expression levels were also upregulated in SA patients (Fig. 5G).

### Circ-CCNB1 Inhibited CCNB1 Nuclear Translocation in HTR-8/SVneo Cells

To explore the effect of circ-CCNB1 on the expression of SIAH1 and CCNB1, SIAH1 and CCNB1 immunostaining was performed following the circ-CCNB1 treatment in HTR-8/SVneo cells. The results demonstrated that overexpression of circ-CCNB1 enhanced the intensity of SIAH1 immunoreactive staining (Fig. 5H) but did not significantly change the intensity of CCNB1 immunoreactive staining (Fig. 5H). However, the CCNB1 expression levels were not significantly changed between the SA patients and normal controls (Supplementary Fig. 1A) (32). The cytoplasmic and nuclear protein expression levels of SIAH1 and CCNB1 were also evaluated after the circ-CCNB1 treatment. The results showed that circ-CCNB1 enhanced SIAH1 cytoplasmic expression and inhibited CCNB1 nuclear protein expression following circ-CCNB1 treatment (Fig. 5I). Furthermore, the cellular localization of SIAH1 was mainly expressed in the cytoplasm and barely changed in the nuclei of in HTR-8/SVneo cells after treatment with circ-CCNB1 (Fig. 5I). However, the circ-CCNB1 treatment suppressed CCNB1 nuclear expression in the HTR-8/SVneo cells (Fig. 5I). Proliferating cell nuclear antigen (PCNA) was used as the internal control for nuclear proteins. These results suggested that circ-CCNB1 inhibited CCNB1 nuclear translocation in HTR-8/SVneo cells.

### Circ-CCNB1 Regulated CCNB1 Nuclear Translocation by Enhancing SIAH1 Expression in Trophoblast Cells

**Figure 6.**
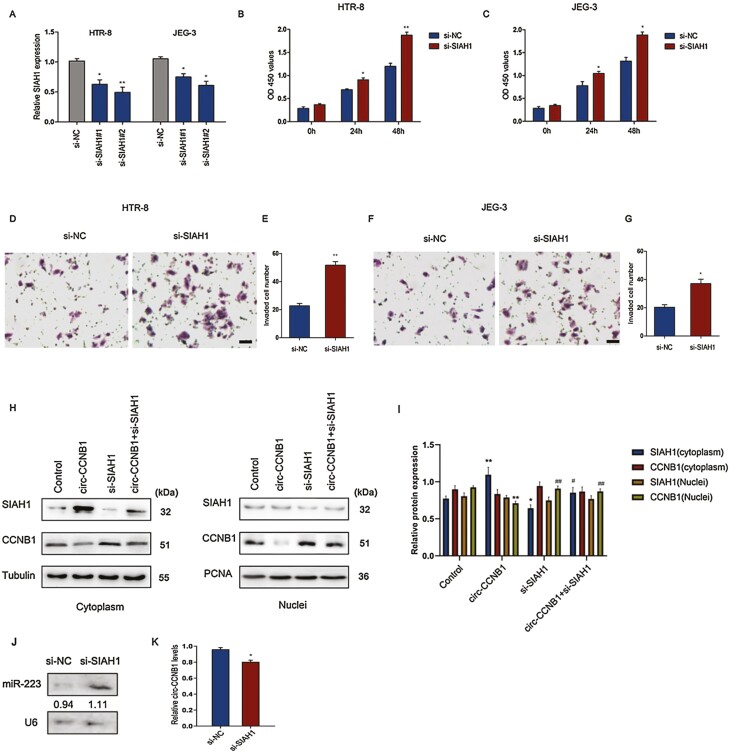
Circ-CCNB1 regulated CCNB1 nuclear translocation by enhancing SIAH1 expression in trophoblast cells. (A) The expression levels of SIAH1 were determined following downregulation of SIAH1 in trophoblast cells. The cell proliferation was estimated by downregulation of SIAH1 for 0, 24, and 48 hours in HTR-8/SVneo (B) or JEG-3 cells (C), *P < 0.05 vs si-NC group, **P < 0.01 VS. si-NC group. The cell invasion was determined by downregulation of SIAH1 for 48 hours in HTR-8/SVneo (D) or JEG-3 cells (F), scale bar: 50 μm. Quantification of the cell invasion in HTR-8/SVneo (E) or JEG-3 cells (G), *P < 0.05, **P < 0.01. The cytoplasmic and nuclear SIAH1 and CCNB1 protein expression levels (H) and quantification of the protein (I) were determined following treatment with circ-CCNB1 and/or si-SIAH1. *P < 0.05, **P < 0.01 vs Control group. ^#^*P* < 0.05, ^##^*P* < 0.01 vs circ-CCNB1 group. (J) Northern blot analysis of miR-223 expression in si-NC or si-SIAH1-treated HTR-8/SVneo cells. (K) The expression levels of circ-CCNB1 were determined in si-NC or si-SIAH1-treated HTR-8/SVneo cells, *P < 0.05. The data were presented for 3 independent experiments. The statistical differences were calculated by 1-way ANOVA analysis of variance with Tukey’s test.

**Figure 7.**
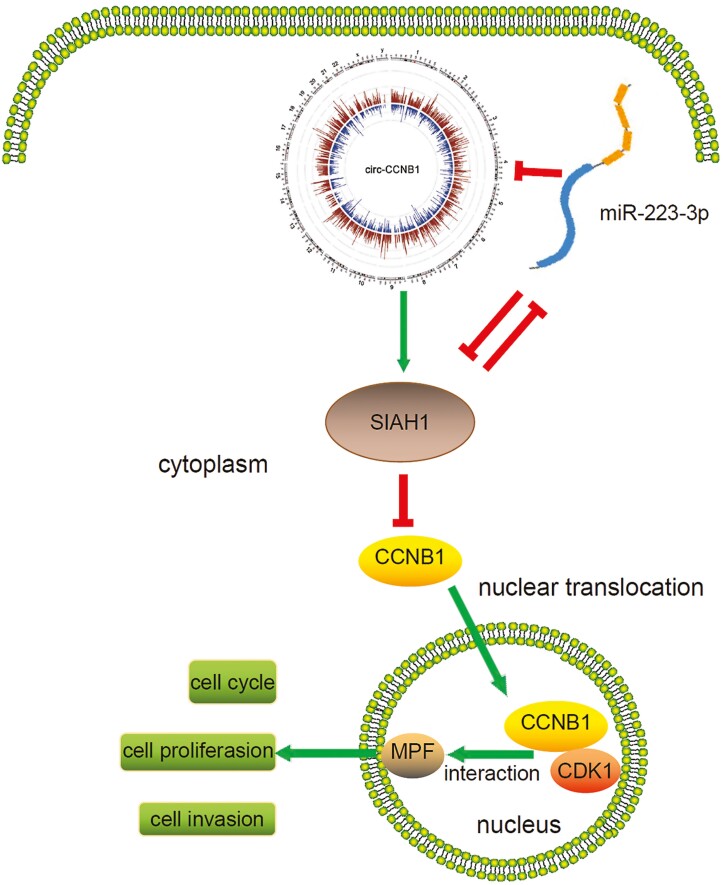
The schematic diagram of the circ-CCNB1/miR-223/SIAH1 axis regulating trophoblast function was depicted in trophoblast cells. Circ-CCNB1 modulates trophoblast proliferation and invasion via sponging miR-223, leading to the activation of SIAH1. Meanwhile, Circ-CCNB1 suppressed CCNB1 nuclear translocation by upregulating the expression of SIAH1 in trophoblast cells. On the other hand, SIAH1 can modulate trophoblast proliferation and invasion through negative feedback regulation of the miR-223/circ-CCNB1 axis. Red arrow indicates inhibition and green arrow indicates promotion. Abbreviations: CDK1, cyclin-dependent kinase 1; MPF, maturation-promoting factor.

To explore the effect of SIAH1 on CCNB1 nuclear translocation, SIAH1 was downregulated by si-SIAH1 treatment in trophoblast cells. The results demonstrated that si-SIAH1#1 and si-SIAH1#2 significantly decreased SIAH1 expression levels in HTR-8/SVneo and JEG-3 cells, respectively (Fig. 6A). To determine the influence of si-SIAH1 on the function of trophoblast cells, cell proliferation was detected in HTR-8/SVneo and JEG-3 cells after transfection with si-SIAH1#2 at the indicated time points. The results showed that downregulation of SIAH1 led to a significant increase in cell proliferation at 24 hours and 48 hours in HTR-8/SVneo and JEG-3 cells compared with those of the si-NC control (Fig. 6B and 6C). Moreover, the invaded cell numbers in the si-SIAH1 treatment group were significantly increased compared with those in the si-NC treatment group in HTR-8/SVneo (Fig. 6D and 6E) and JEG-3 (Fig. 6F and 6G) cells. In addition, downregulation of SIAH1 significantly enhanced circ-CCNB1-suppressed CCNB1 nuclear protein expression (Fig. 6H and 6I). The latest studies have shown that E3 ubiquitin ligases can promote miRNA degradation and mediate target-directed miRNA degradation (TDMD) (34, 35). To investigate the potential feedback regulation of the miR-223/circ-CCNB1 axis by SIAH1 E3 ubiquitin, we determined the miR-233 and circ-CCNB1 expression levels following the downregulation of SIAH1. Intriguingly, we found that downregulation of SIAH1 suppressed the degradation of miR-223 and the expression levels of circ-CCNB1, while enhanced the CCNB1 levels in HTR-8/SVneo cells (Fig. 6J and 6K, Supplementary Fig. 1B) (32), suggesting that SIAH1 E3 ubiquitin ligases might modulate trophoblast proliferation and invasion through negative feedback regulation of the miR-223/circ-CCNB1 axis. Together, these results demonstrated that circ-CCNB1 plays a role in suppressing trophoblast proliferation and invasion via the miR-223/SIAH1 pathway during embryo implantation (Fig. 7).

## Discussion

In the present study, we found that circ-CCNB1, a regulatory circRNA involved in the cell cycle, was upregulated in villous tissues of patients with SA. We found that after transfection with circ-CCNB1 overexpression vectors, the proliferation and invasion abilities of HTR-8/SVneo and JEG-3 cells were significantly suppressed. Overexpression of circ-CCNB1 led to a significant increase in the percentage of HTR-8/SVneo cells in the G0/G1 phase and a significant decrease in the percentage in the S phase. Then, we verified that circ-CCNB1 suppressed trophoblast cellular proliferation and invasion partly by sponging miR-223. In addition, we determined that miR-223 targeted SIAH1 and reversed the inhibition of CCNB1 nuclear translocation induced by circ-CCNB1 in trophoblast cells, thus forming a novel network between the target molecule CCNB1 and the corresponding circ-CCNB1.

JEG3 is a choriocarcinoma, and HTR8/SVneo is an extravillous trophoblast cell line. JEG-3 and HTR8/SVneo cells have been widely used as a model for placental trophoblast, as well as modeling the physiologically invasive extravillous trophoblast in spontaneous abortion and early pregnancy (36, 37). CircRNAs can regulate gene expression by influencing transcription, mRNA turnover, and translation by sponging RNA-binding proteins and microRNAs (38). Numerous abundant circRNAs are specifically expressed in various tissues and differentially expressed in cancer compared with normal tissues, which suggests that these RNAs serve a specific function in these tissues. Recent results suggest that circ-CCNB1 is a powerful circular RNA in the inhibition of tumor progression (34). However, whether and how circ-CCNB1 acts during the process of embryo implantation at the maternal-fetal interface by regulating trophoblast cell proliferation and invasion is still unknown. circ-CCNB1 has been reported to inhibit breast cancer cell proliferation and invasion (31). In the present study, the expression levels of circ-CCNB1 in SA patients were significantly increased compared with those in normal controls.

To further understand the biological role of circ-CCNB1, the regulatory network in which the associated miRNAs or proteins participate was considered. CircRNAs have been shown to act as microRNA (miRNA) sponges to regulate target genes in a variety of pregnancy-related diseases (39, 40). In our study, the bioinformatics analysis showed that miR-370 and miR-223 were the overlapping miRNAs targeted by circ-CCNB1. Only miR-223 was significantly suppressed after upregulation of circ-CCNB1. Furthermore, the luciferase reporter and pull-down assay results verified the interaction between circ-CCNB1 and miR-223. A recent study reported that miR-223 expression levels were significantly decreased in patients with recurrent pregnancy loss compared with normal controls (33). Consistently, our study also showed that the levels of miR-223 in patients with SA were significantly lower than those in normal controls, which was negatively associated with circ-CCNB1 levels. Herein, we showed that circ-CCNB1 could serve as a modulator of cell growth partly by sponging miR-223 in trophoblast cells during early pregnancy.

Studies have demonstrated that miRNA expression signatures are highly tissue- and disease specific. The miR-223-encoding gene is located within the q12 locus of the X chromosome, and its sequence is highly conserved during evolution, suggesting its potential role in essential physiological events (41). Previous studies revealed that miR-223 expression levels were downregulated in human preeclampsia placentas (42, 43). Additionally, miR-223 levels were reported to be downregulated in patients with recurrent pregnancy loss (33), thus highlighting the important role of miR-223 in embryo implantation. In the present study, we found that miR-223 inhibitor treatment led to a significant decrease in the proliferation and invasion of trophoblast cells, meaning that downregulation of miR-223 could potentially impair the functions of trophoblast cells. Taken together, a dynamic balance in the expression levels and interaction between miR-223 and circ-CCNB1 in trophoblast cells at the maternal-fetal interface in early pregnancy should be maintained to ensure the normal function of proliferation and invasion and thus represents a prerequisite for normal embryo implantation. If abnormal expression levels or interactions are observed between miR-223 and circ-CCNB1, then impaired proliferation and invasion occur, which will lead to abnormal embryo implantation and corresponding pregnancy-related diseases, such as SA.

MiR-223 could alter different downstream targets in various cell types; hence, investigating the gene targets of miR-223-regulated trophoblast proliferation and invasion may help us to elucidate the molecular mechanisms involved in normal embryo implantation or in SA. Using bioinformatics tools, we analyzed the miR-223 potential targeted cyclin-related genes, ubiquitin ligases, transcription factors, and apoptotic factors. MiR-223 dramatically increased the expression levels of cyclin-related genes, such as CCNB1, TK1, KLF4 and the transcription factor NF-κB, but significantly decreased the expression levels of the E3 ubiquitin ligases SIAH1 and FBXW7 and the apoptotic factor caspase-3. A previous study showed that CacyBP/SIP inhibited the migration and invasion of glioblastoma cells by activating the SIAH1-mediated ubiquitination and degradation of cytoplasmic p27 (44). MiR-107 has been shown to promote human breast cancer cell proliferation, colony formation, migration and invasion and inhibit apoptosis by reducing SIAH1 expression (28). In the present study, we found that miR-223 directly targeted SIAH1 and that overexpression of miR-223 decreased circ-CCNB1-enhanced SIAH1 expression levels in HTR-8/SVneo cells, indicating that miR-223 targeted SIAH1 and reversed circ-CCNB1-enhanced SIAH1 expression in HTR-8/SVneo cells.

Upregulation of circ-CCNB1 may suppress trophoblast proliferation and invasion through inhibition of CCNB1 nuclear translocation partly by enhancing SIAH1 expression. CCNB1 is mainly distributed in the cytoplasm during mitosis interphase but accumulates in the nucleus to promote chromosome condensation. In addition, CCNB1 initiates mitotic spindle assembly during mitosis prometaphase through interaction with cyclin-dependent kinase 1 (CDK1) in the nucleus, which is essential for cell cycle progression through mitosis (45). Knockout of CCNB1 inhibited cell proliferation in vitro and in vivo (46, 47), whereas overexpression of CCNB1 or CDK1 could promote cell division in postmitotic mouse, rat, and human cardiomyocytes (48). In tumor cells, circ-CCNB1 has been shown to bind to both CCNB1 and CDK1, thereby inhibiting the formation of the CCNB1-CDK1 complex and its entry into the nucleus, resulting in the inhibition of tumor growth and extension of mouse viability. However, in our study, we found a supplementary pathway of circ-CCNB1 in modulating CCNB1 nuclear translocation, suggesting that the promotion of proliferation and invasion in trophoblast cells requires cells to coordinate multiple mechanisms to achieve a dynamic balance. Moreover, even in the same regulatory pathway, circ-CCNB1 may simultaneously interact with miR-223 as well as with CCNB1 and CDK1 (22). Furthermore, miR-223 might mediate the ubiquitination and degradation of CCNB1 by interacting with SIAH1, although further investigations are required to verify this hypothesis.

In summary, we found for the first time that circ-CCNB1 was significantly upregulated in villous tissues from patients with SA and might serve as a modulator of cell growth by sponging miR-223, thereby forming a network circled by circ-CCNB1/miR-223/SIAH1 in modulating CCNB1 nuclear translocation. These results help us to elucidate the molecular mechanisms involved in normal embryo implantation or in SA, although the actual implications of these pathways in the diagnosis or treatment of SA have yet to be determined.

## Data Availability

Some or all datasets generated during and/or analyzed during the current study are not publicly available but are available from the corresponding author on reasonable request.

## Abbreviations

bio: biotin
BrdU: bromodeoxyuridine
CCK-8: cell counting kit-8
circ-CCNB1: circular RNA cyclin B1
CDK1: cyclin dependent kinase 1
circRNA: circular RNA
DAPI: 4′,6-diamidino-2-phenylindole
FISH: fluorescence in situ hybridization
GFP: green fluorescent protein
miRNA: microRNA
mut: mutant
NC: negative control
PBS: phosphate-buffered saline
qRT-PCR: quantitative reverse-transcriptase polymerase chain reaction
SA: spontaneous abortion
SIAH1: seven in absentia homolog-1
WT: wild-type

## References

[1] Alves C , Rapp A. Spontaneous abortion. In: StatPearls. Treasure Island (FL): StatPearls Publishing; May 1, 2022.

[2] Mohammadi S , Abdollahi E, Nezamnia M, et al Adoptive transfer of Tregs: a novel strategy for cell-based immunotherapy in spontaneous abortion: lessons from experimental models. Int Immunopharmacol. 2021;90:107195. doi:10.1016/j.intimp.2020.107195 33278746

[3] Sundermann AC , Velez Edwards DR, Bray MJ, Jones SH, Latham SM, Hartmann KE. Leiomyomas in pregnancy and spontaneous abortion: a systematic review and meta-analysis. Obstet Gynecol. 2017;130:1065-1072. doi:10.1097/AOG.0000000000002313 29016496 PMC5656535

[4] Muyayalo KP , Li ZH, Mor G, Liao AH. Modulatory effect of intravenous immunoglobulin on Th17/Treg cell balance in women with unexplained recurrent spontaneous abortion. Am J Reprod Immunol. 2018;80(4):e13018. doi:10.1111/aji.13018 29984444

[5] Yang F , Zheng Q, Jin L. Dynamic function and composition changes of immune cells during normal and pathological pregnancy at the maternal-fetal interface. Front Immunol. 2019;10:2317. 31681264 10.3389/fimmu.2019.02317 PMC6813251

[6] Jena MK , Nayak N, Chen K, Nayak NR. Role of macrophages in pregnancy and related complications. Arch Immunol Ther Exp (Warsz). 2019;67(5):295-309. doi:10.1007/s00005-019-00552-7 31286151 PMC7140981

[7] Zhang Y , Zhou J, Li MQ, Xu J, Zhang JP, Jin LP. MicroRNA-184 promotes apoptosis of trophoblast cells via targeting WIG1 and induces early spontaneous abortion. Cell Death Dis. 2019;10(3):223. doi:10.1038/s41419-019-1443-2 30833572 PMC6399231

[8] Wu L , Cheng B, Liu Q, Jiang P, Yang J. CRY2 suppresses trophoblast migration and invasion in recurrent spontaneous abortion. J Biochem. 2020;167(1):79-87. doi:10.1093/jb/mvz076 31536114

[9] Liu HN , Tang XM, Wang XQ, et al MiR-93 inhibits trophoblast cell proliferation and promotes cell apoptosis by targeting BCL2L2 in recurrent spontaneous abortion. Reprod Sci. 2020;27:152-162. doi:10.1007/s43032-019-00003-w 32046397

[10] Ala U . Competing endogenous RNAs, non-coding RNAs and diseases: an intertwined story. Cells. 2020;9:1574. doi:10.3390/cells9071574 PMC7407898 32605220

[11] Liu CJ , Fu X, Xia M, Zhang Q, Gu Z, Guo AY. miRNASNP-v3: a comprehensive database for SNPs and disease-related variations in miRNAs and miRNA targets. Nucleic Acids Res. 2021;49:D1276-D1281. doi:10.1093/nar/gkaa783 32990748 PMC7778889

[12] Haneklaus M , Gerlic M, O’Neill LA, Masters SL. miR-223: infection, inflammation and cancer. J Intern Med. 2013;274:215-226. doi:10.1111/joim.12099 23772809 PMC7166861

[13] Ma J , Cao T, Cui Y, et al miR-223 regulates cell proliferation and invasion via targeting PDS5B in pancreatic cancer cells. Mol Ther Nucleic Acids. 2019;14:583-592. doi:10.1016/j.omtn.2019.01.009. 30776580 PMC6378631

[14] Ji Q , Xu X, Song Q, et al miR-223-3p inhibits human osteosarcoma metastasis and progression by directly targeting CDH6. Mol Ther. 2018;26:1299-1312. doi:10.1016/j.ymthe.2018.03.009 29628305 PMC5993963

[15] Dong X , Kong C, Liu X, et al GAS5 functions as a ceRNA to regulate hZIP1 expression by sponging miR-223 in clear cell renal cell carcinoma. Am J Cancer Res. 2018;8:1414-1426. 30210913 PMC6129482

[16] Fang C , Xu L, He W, Dai J, Sun F. Long noncoding RNA DLX6-AS1 promotes cell growth and invasiveness in bladder cancer via modulating the miR-223-HSP90B1 axis. Cell Cycle. 2019;18:3288-3299. doi:10.1080/15384101.2019.1673633 31615303 PMC6927722

[17] Patop IL , Wust S, Kadener S. Past, present, and future of circRNAs. EMBO J. 2019;38:e100836. doi:10.15252/embj.2018100836 31343080 PMC6694216

[18] Das A , Gorospe M, Panda AC. The coding potential of circRNAs. Aging. 2018;10:2228-2229. doi:10.18632/aging.101554 30215602 PMC6188482

[19] Conn SJ , Pillman KA, Toubia J, et al The RNA binding protein quaking regulates formation of circRNAs. Cell. 2015;160:1125-1134. doi:10.1016/j.cell.2015.02.014 25768908

[20] Dang Y , Yan L, Hu B, et al Tracing the expression of circular RNAs in human pre-implantation embryos. Genome Biol. 2016;17:130. doi:10.1186/s13059-016-0991-3 27315811 PMC4911693

[21] Zhang S , Ding Y, He J, et al Altered expression patterns of circular RNAs between implantation sites and interimplantation sites in early pregnant mice. J Cell Physiol. 2019;234:9862-9872. doi:10.1002/jcp.27675 30370529

[22] Fang L , Du WW, Awan FM, Dong J, Yang BB. The circular RNA circ-Ccnb1 dissociates Ccnb1/Cdk1 complex suppressing cell invasion and tumorigenesis. Cancer Lett. 2019;459:216-226. 31199987 10.1016/j.canlet.2019.05.036

[23] Yuan J , Kramer A, Matthess Y, et al Stable gene silencing of cyclin B1 in tumor cells increases susceptibility to taxol and leads to growth arrest in vivo. Oncogene. 2006;25:1753-1762. doi:10.1038/sj.onc.1209202 16278675

[24] Mashal RD , Lester S, Corless C, et al Expression of cell cycle-regulated proteins in prostate cancer. Cancer Res. 1996;56:4159-4163. 8797586

[25] Yu D , Liu R, Yang G, Zhou Q. The PARP1-Siah1 axis controls HIV-1 transcription and expression of siah1 substrates. Cell Rep. 2018;23:3741-3749. doi:10.1016/j.celrep.2018.05.084 29949759 PMC6223328

[26] Ko HR , Jin EJ, Lee SB, et al SIAH1 ubiquitin ligase mediates ubiquitination and degradation of Akt3 in neural development. J Biol Chem. 2019;294:15435-15445. doi:10.1074/jbc.RA119.009618 31471318 PMC6802513

[27] Deng Q , Hou J, Feng L, et al PHF19 promotes the proliferation, migration, and chemosensitivity of glioblastoma to doxorubicin through modulation of the SIAH1/beta-catenin axis. Cell Death Dis. 2018;9:1049. doi:10.1038/s41419-018-1082-z 30323224 PMC6189144

[28] Zhang L , Ma P, Sun LM, et al MiR-107 down-regulates SIAH1 expression in human breast cancer cells and silencing of miR-107 inhibits tumor growth in a nude mouse model of triple-negative breast cancer. Mol Carcinog. 2016;55:768-777. doi:10.1002/mc.22320 25851994

[29] He WH , Jin MM, Liu AP, et al Estradiol promotes trophoblast viability and invasion by activating SGK1. Biomed Pharmacother. 2019;117:109092. doi:10.1016/j.biopha.2019.109092 31203134

[30] Su S , Zhao Q, He C, et al miR-142-5p and miR-130a-3p are regulated by IL-4 and IL-13 and control profibrogenic macrophage program. Nat Commun. 2015;6:8523. doi:10.1038/ncomms9523 26436920 PMC4600756

[31] Fang L , Du WW, Lyu J, et al Enhanced breast cancer progression by mutant p53 is inhibited by the circular RNA circ-Ccnb1. Cell Death Differ. 2018;25:2195-2208. doi:10.1038/s41418-018-0115-6 29795334 PMC6261950

[32] Liu A , Wang D. Supplemental Figure: Circ-CCNB1 modulates trophoblast proliferation and invasion in spontaneous abortion by regulating miR-223/SIAH1 axis, Dryad, Dataset. April 8, 2022; 10.5061/dryad.1zcrjdftx. PMC9290912 35731831

[33] Azizi R , Soltani-Zangbar MS, Sheikhansari G, et al Metabolic syndrome mediates inflammatory and oxidative stress responses in patients with recurrent pregnancy loss. J Reprod Immunol. 2019;133:18-26. doi:10.1016/j.jri.2019.05.001. 31100644

[34] Han J , LaVigne CA, Jones BT, Zhang H, Gillett F, Mendell JT. A ubiquitin ligase mediates target-directed microRNA decay independently of tailing and trimming. Science. 2020;370:eabc9546. 33184234 10.1126/science.abc9546 PMC8177725

[35] Shi CY , Kingston ER, Kleaveland B, Lin DH, Stubna MW, Bartel DP. The ZSWIM8 ubiquitin ligase mediates target-directed microRNA degradation. Science. 2020;370:eabc9359. 33184237 10.1126/science.abc9359 PMC8356967

[36] Wang XH , Xu S, Zhou XY, et al Low chorionic villous succinate accumulation associates with recurrent spontaneous abortion risk. Nat Commun. 2021;12(1):3428. doi:10.1038/s41467-021-23827-0 34103526 PMC8187647

[37] Wang B , Xu T, Li Y, et al Trophoblast H2S maintains early pregnancy via regulating maternal-fetal interface immune hemostasis. J Clin Endocrinol Metab. 2020;105(12):e4275-e4289. 10.1210/clinem/dgaa357 PMC7526739 32506120

[38] Panda AC . Circular RNAs act as miRNA sponges. Circular RNAs act as miRNA sponges. Adv Exp Med Biol. 2018;1087:67-79. doi:10.1007/978-981-13-1426-1_6 30259358

[39] Nagy B . Cell-free nucleic acids in prenatal diagnosis and pregnancy-associated diseases. Ejifcc. 2019;30:215-223. 31263394 PMC6599189

[40] Jia N , Li J. Role of circular RNAs in preeclampsia. Dis Markers. 2019;2019:7237495. doi:10.1155/2019/7237495. 31191755 PMC6525895

[41] Ye D , Zhang T, Lou G, Liu Y. Role of miR-223 in the pathophysiology of liver diseases. Exp Mol Med. 2018;50:1128-1112. doi:10.1038/s12276-018-0153-7. PMC6158210 30258086

[42] Xu P , Zhao Y, Liu M, et al Variations of microRNAs in human placentas and plasma from preeclamptic pregnancy. Hypertension. 2014;63:1276-1284. doi:10.1161/HYPERTENSIONAHA.113.02647 24664294

[43] Weedon-Fekjær MS , Sheng Y, Sugulle M, et al Placental miR-1301 is dysregulated in early-onset preeclampsia and inversely correlated with maternal circulating leptin. Placenta. 2014;35:709-717. doi:10.1016/j.placenta.2014.07.002 25064070

[44] Yan S , Li A, Liu Y. CacyBP/SIP inhibits the migration and invasion behaviors of glioblastoma cells through activating Siah1 mediated ubiquitination and degradation of cytoplasmic p27. Cell Biol Int. 2018;42:216-226. 29024247 10.1002/cbin.10889

[45] Gavet O , Pines J. Progressive activation of CyclinB1-Cdk1 coordinates entry to mitosis. Dev Cell. 2010;18:533-543. doi:10.1016/j.devcel.2010.02.013 20412769 PMC3325599

[46] Zhang H , Zhang X, Li X, et al Effect of CCNB1 silencing on cell cycle, senescence, and apoptosis through the p53 signaling pathway in pancreatic cancer. J Cell Physiol. 2018;234:619-631. doi:10.1002/jcp.26816. 30069972

[47] Brandeis M , Rosewell I, Carrington M, et al Cyclin B2-null mice develop normally and are fertile whereas cyclin B1-null mice die in utero. Proc Natl Acad Sci USA. 1998;95:4344-4349. doi:10.1073/pnas.95.8.4344 9539739 PMC22491

[48] Mohamed TMA , Ang YS, Radzinsky E, et al Regulation of cell cycle to stimulate adult cardiomyocyte proliferation and cardiac regeneration. Cell. 2018;173:104-116.e12. doi:10.1016/j.cell.2018.02.014 29502971 PMC5973786

